# FLAVi: An Enhanced Annotator for Viral Genomes of *Flaviviridae*

**DOI:** 10.3390/v12080892

**Published:** 2020-08-14

**Authors:** Adriano de Bernadi Schneider, Denis Jacob Machado, Sayal Guirales, Daniel A. Janies

**Affiliations:** 1 AntiViral Research Center, Department of Medicine, University of California San Diego, San Diego, CA 92103, USA; adeberna@ucsd.edu; 2 Department of Bioinformatics and Genomics, College of Computing and Informatics, University of North Carolina at Charlotte, Charlotte, NC 28223, USA; sguirale@uncc.edu (S.G.); djanies@uncc.edu (D.A.J.)

**Keywords:** viral evolution, arbovirus, phylogenomics, gene annotation

## Abstract

Responding to the ongoing and severe public health threat of viruses of the family *Flaviviridae*, including dengue, hepatitis C, West Nile, yellow fever, and Zika, demands a greater understanding of how these viruses emerge and spread. Updated phylogenies are central to this understanding. Most cladograms of *Flaviviridae* focus on specific lineages and ignore outgroups, hampering the efficacy of the analysis to test ingroup monophyly and relationships. This is due to the lack of annotated *Flaviviridae* genomes, which has gene content variation among genera. This variation makes analysis without partitioning difficult. Therefore, we developed an annotation pipeline for the genera of *Flaviviridae* (*Flavirirus*, *Hepacivirus*, *Pegivirus*, and *Pestivirus*, named “Fast Loci Annotation of Viruses” (FLAVi; http://flavi-web.com/), that combines ab initio and homology-based strategies. FLAVi recovered 100% of the genes in *Flavivirus* and *Hepacivirus* genomes. In *Pegivirus* and *Pestivirus*, annotation efficiency was 100% except for one partition each. There were no false positives. The combined phylogenetic analysis of multiple genes made possible by annotation has clear impacts over the tree topology compared to phylogenies that we inferred without outgroups or data partitioning. The final tree is largely congruent with previous hypotheses and adds evidence supporting the close phylogenetic relationship between dengue and Zika.

## 1. Introduction

The family *Flaviviridae* comprises the genera *Flavivirus*, *Hepacivirus*, *Pegivirus*, and *Pestivirus*, all of which share structural and genomic similarity [1]. There are several examples that demonstrate the clinical relevance and zoonotic nature of this family. In humans, hepatitis C virus (HCV) causes a disease that damages the liver [2]. Human pegivirus-1 (HPgV-1), causes human encephalitis [3] as well bovine viral diarrhea (BVDV), a long-known cause of major losses in livestock [4]. *Flaviviridae* also houses viruses known to cause neglected tropical diseases of the genus *Flavivirus*, which comprises over 100 different pathogens including notable viruses such as dengue (DENV), West Nile (WNV), yellow fever (YFV), and Zika (ZIKV) [5]. Note that the International Committee on Taxonomy of Viruses code (ICTV; http://ictv.global/code) indicates that the first letters of words in a virus name should be capitalized if they are proper nouns. However, many authors capitalize Zika virus, assuming that Zika is a proper noun. We are replicating this common practice here even though the proper noun would be Ziika, with double i, from the Ziika Forest.

*Flaviviridae* have linear, single-stranded positive RNA genomes of 9–13 kb. These genomes contain a single polyprotein, divided into structural and non-structural genes, and flanked by a 3’ and a 5’ untranslated region (UTR).

The combination of the information from ViralZone [6] and the annotated genomes of *Flaviviridae* from the National Center for Biotechnology Information Reference Sequence Database (NCBI’s RefSeq) indicate that a NS5 gene, composed of NS5A and RNA-dependent RNA polymerase (RdRp), is common to the four genera.

However, not all *Flaviviridae* code the same proteins. The genome of *Flavivirus* consists of three structural (C, prM, E), and eight non-structural (NS1, NS2A, NS2B, NS3, NS4A, 2k, NS4B, NS5) proteins. Three of the proteins of *Flaviviridae* are exclusive (prM, NS1, and 2k) to the family [1]. *Hepacivirus* contains two envelope proteins (E1 and E2) while *Pestivirus* contains three (Erns, E1, and E2). The E of *Flavivirus* is equivalent to the E1-E2 dimer in the other genera. *Pegivirus* lacks the capsid protein (C) which is present in the other three genera. It is not clear if an additional C might arise in the genus from cleavage of the N-terminus or from an alternative reading frame. Finally, a Npro protease precedes the C in *Pestivirus*.

Given the medical and economic significance of *Flaviviridae*, there is an increasing interest in understanding their genomic structure and evolution as well as comparing known viruses of this family with less known viruses that may become a health concern in the upcoming years. At the time of writing this report, there were 8633 complete genomes of *Flaviviridae* available in NCBI’s GenBank and RefSeq databases [7]. RefSeq sequences are not part of the International Nucleotide Sequence Database Collaboration (INSDC), but are derived from INSDC sequences to provide non-redundant curated data representing our current knowledge of known genes. Some records include sequence information gathered from more than one INSDC record. We avoided redundancy by excluding GenBank sequences that were present in our RefSeq selection. Only 5217 (approx. 60%) of the 8633 complete genomes from RefSeq and GenBank were annotated beyond the polyprotein level. This lack of genome annotation thwarts comparative analyses at the level of individual proteins.

A review of 58 articles on *Flaviviridae* published since 2018 citing records of complete genomes of this family in GenBank and RefSeq shows that most of these authors analyze the entire polyprotein sequences with no granular annotation or data partitioning of any kind. Authors who annotate genes within the viral polyprotein are scarce and perform the annotations by using a variety of methods. As such, the annotations are absent or do not follow a consistent methodology. For example, Charles et al. [8] identified conserved domains utilizing blastn and blastp [9]. Wu et al. [10] deduced polyproteins on pestiviruses by aligning them to other known sequences of the genus and predicted conserved protein domains using either Pfam [11] and InterProScan [12] or the Conserved Domain Database of NCBI [13]. As a final example, Wen et al. [14] predicted coding regions using the Predict Protein Server [15].

Our goal of maximizing the number of fully and consistently annotated genomes of *Flaviviridae* allows us to analyze homologous sequences of proteins of interest individually or in combination with other data. A complete gene dataset for *Flaviviridae* will enable phylogeneticists to include various genera in a single analysis since one is then able to partition the data taking into account genes that are not ubiquitous in the family. Without sequence annotation to guide data partitioning, the variation of gene content among different genera make it impossible to include all their genomes in the same analysis. Moreover, the alignment of complete polyproteins, even of closely related flaviviruses, can lead to false homology assumptions in which nucleotides of one gene align with nucleotides of another. Therefore, partitioning is key for a group in which phylogenies frequently include only members of a single genus (using no outgroup sequences).

In the absence of outgroup sequences, many authors resort to strategies such as midpoint rooting (e.g., [16,17,18]). Many authors argue that the method is valuable, particularly for cases where a proper outgroup is unavailable, but can be less reliable the more inconsistent (for outgroup root consistency checks, see [19]) the outgroup root is [20]. Also, most midpoint rooting algorithms require an equal molecular clock in all lineages, and that is highly unlikely [21]. There are other caveats in not using an outgroup (for example, see [22]). Without outgroup comparison, we can test hypotheses of ingroup topology, but not its monophyly. We can also test the hypotheses of homology without reference to other taxa’s character states, but we cannot evaluate the homologies of the entire ingroup. Finally, the inclusion of additional taxa often increases the severity of the test and the inclusion of outgroup taxa will clarify the phylogenetic relationships within the ingroup if the analysis is unconstrained [23].

To overcome these shortcomings, we developed a new approach to annotate genomes from any of the four genera of *Flaviviridae* and evaluated its potential impact on the phylogenetic analyses of these viruses. It has not escaped our notice that there are authors (e.g., [17,24,25]) that have expressed concern regarding the combination of different genera or even species of *Flaviviridae* into the same phylogeny fearing that it could lead to long-branch attraction (LBA) events in which the outgroup would be attracted to the largest ingroup branch. Hence, we also test and discuss LBA events that could arise in such a scenario and present an argument in favor of rooting on outgroup sequences instead of applying midpoint rooting in viral phylogenetics.

## 2. Materials and Methods

### 2.1. Taxon Sampling

We downloaded 78 complete reference genome sequences of *Flaviviridae* from NCBI’s Reference Sequence (RefSeq) database (https://www.ncbi.nlm.nih.gov/refseq/) and 41 complete genome sequences of *Flaviviridae* from NCBI’s GenBank (https://www.ncbi.nlm.nih.gov/genbank/). These 119 sequences include 103 complete viral genomes that we could unambiguously assign to the genus *Flavivirus* at the moment of download. These sequences additionally comprise four genomes of *Pestivirus*, five genomes of *Pegivirus*, and seven genomes of *Hepacivirus*. The total nucleotide count of the data is 1.27 Mbp (with an average or 10,663 bases per genome). Note that not all RefSeq sequences contained a complete list of annotated genes. In total, only 63 RefSeq genomes were fully annotated (48 *Flavivirus*, seven *Hepacivirus*, four *Pegivirus*, and four *Pestivirus*). See Table S1 (Supplementary Materials).

With taxon selection we do not aim to encompass all available taxa within the *Flaviviridae* family. Instead, we built datasets to demonstrate the effectiveness of the proposed pipeline and the importance of gene annotation and outgroup selection in recovering the ingroup topology, composed of *Flavivirus* genomes.

We selected *Flavivirus* as an ingroup due to its high diversity within our taxon sampling. A diverse ingroup is more likely to reveal insights into topology changes due to the influence of outgroup selection and data partitioning.

### 2.2. New Approaches for Gene Prediction in Flaviviruses

Here, we present a new gene annotation pipeline, named “Fast Loci Annotation of Viruses” (FLAVi). This tool is dedicated to annotate genomes of the family *Flaviviridae* and takes into account their specific characteristics (i.e., a linear and positive-sense single-stranded RNA that codes genomic polyprotein in which genes are not necessarily flanked by start and stop-codons).

Each genomic sequence of *Flaviviridae* was run through a pipeline that started with parallel GeneWise (comes with Wise v2.4.1 [26] and TransDecoder v.3.0.0 [27]) analyses. In TransDecoder, we used dedicated databases downloaded from UniProt [28] and blastp (comes with BLAST v2.4.0+ [9]) for homology-based annotation. The user may decide to change or update the gene databases used for homology-based annotation. These databases that come with FLAVi consider all the diversity in gene content among the different genera of *Flaviviridae*.

The pipeline also executes ab initio analyses for different genes simultaneously, allowing the user to leverage on multiple processors. It allows the user to easily update datasets for homology searches and to train new gene models. We employed hmmscan (comes with HMMER v3.1b2, available at http://hmmer.org/) to search the peptides for protein domains using Pfam [11]. If predictions from both GeneWise and TransDecoder matched, we pooled the alignments and calculated distance matrices with distmat (comes with EMBOSS v6.6.0, [29]).

**Figure 1:**
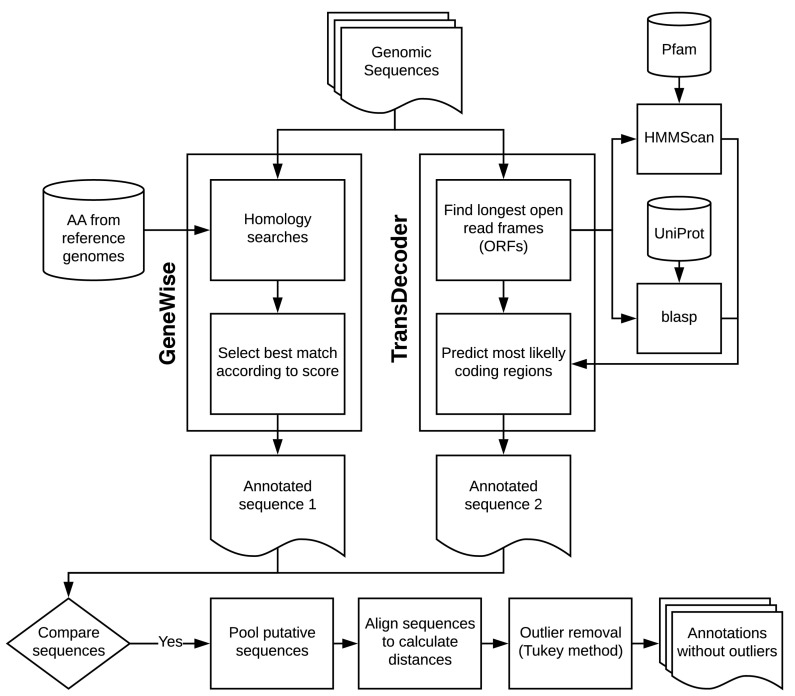
Main steps of the protein annotation pipeline.

Finally, we applied the distance matrices for outlier testing using the Tukey method [30] with the help of original R scripts (see Results). The Tukey method of searching for outliers leverages on the interquartile range and is applicable to most ranges since it is not dependent on distributional assumptions. It also ignores the mean and standard deviation, making it resistant to being influenced by the extreme values in the range. We removed all sequences that were selected as outliers. See  Figure 1 for a description of the main steps of this pipeline.

### 2.3. Gene Partitions

**Figure 2:**
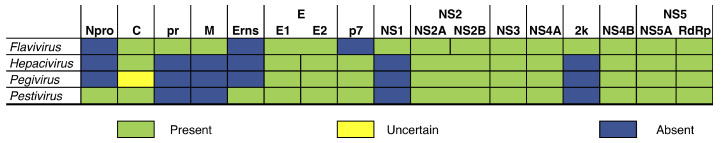
Genome structure comparison between the four genera of *Flaviviridae* (*Flavivirus*, *Hepacivirus*, *Pegivirus* and *Pestivirus*), showing the 14 partitions that can be found in the genomes of the family. The columns on this table are not proportional to partition lengths.

Combined, we can divide the polyproteins of the four genera of *Flaviviridae* into 14 protein regions, although not all regions are common to the entire family (Figure 2). To understand the effects of data partitioning and outgroup sampling on the phylogenetic relationships of *Flaviviridae*, we used three different strategies to partition the data for the phylogenetic analysis based on genome annotation. First, we used a single partition for the entire coding region for each polyprotein in *Flavivirus*. Second, we used 11 partitions for the analysis of sequences of *Flavivirus* (i.e., all partitions except Npro, Erns, and p7). Lastly, we used 14 partitions including all four genera, in which we coded absent genes as missing data. Although partitioning was based on different proteins, multiple sequence alignments (MSAs) and phylogenetic analyses are based on the nucleotide sequences of each partition, not amino acids.

### 2.4. Multiple Sequence Alignment Test

To test the hypothesis that both amino acid and nucleotide alignments will often lead to molecules from different genes aligning together as different character states of the same character (henceforth called “gene misalignment”). To perform this test we used Clustal Omega [31] to produce the MSAs of nucleotide and amino acid sequences of *Flaviviridae* polyproteins and counted the total number of shared genes shared among genomes as well as the number of misaligned genes. For this test, we selected reference and annotated sequences belonging to the genus *Flavivirus* found in three well-defined clades of this genus: Clade 1: *Aedes* spp. associated MBV I (NCBI’s accession nos. NC_027999, NC_002031, NC_012735, NC_027700, NC_008719, and NC_030289); Clade 2: *Aedes* spp. associated MBV II (NC_001475, NC_001477, NC_001474, NC_002640, NC_012532, NC_029055, and NC_012533); and Clade 3: ISFV-like I (NC_024806, NC_017086, NC_024805, NC_016997). We also aligned sequences of a set of diverse flaviviruses from different clades (NC_001477, NC_016997, NC_002031, NC_001809, NC_009942).

### 2.5. Tree Search

We applied the following MSA techniques to account for the sensitivity of tree topology to the variation on homology statements at the character level: MAFFT, Clustal, MUSCLE, and the Geneious’ translation-based alignment tool under different scoring matrices (PAM100, PAM200, and PAM250). We used Geneious (v8.1.9) [32] to execute every alignment using default arguments. Our criteria for selection of best alignment strategies was the maximization of overall sequence identity and minimization of alignment length (i.e., if sequence identity was the same, the shortest alignment would be selected).

To focus on the effects of data partitioning and to minimize possible artifacts caused by missing data on partitions that are not found in all *Flaviviridae*, we built datasets with (1) nucleotide sequences of complete polyproteins aligned without partitioning and (2) partitioned nucleotide sequences of proteins of flaviviruses that contained all partitions. To address the sensitivity of tree topology to the inclusion of outgroup sequences, we used partitioned data on analyses containing either exclusively flaviviruses or all the four genera of its family. Finally, since tree search can be affected by the choice of optimality criterion, we explored the results of three phylogenetic tree search methodologies: TNT 64-bit version with no taxon limit (parsimony—P) [33], IQ-Tree v1.6.1 (maximum likelihood—ML) [34] and BEAST v2.4.8 (Bayesian inference—BI, under the criterion of posterior probability—PP) [35]. See Table S3 and File S2 (Supplementary Materials) for non-default arguments used for each tree search and the configuration file used for BEAST, respectively.

In total, we performed 31 tree search analyses considering different alignments and optimality criteria. Of those, ten trees are from non-partitioned alignments of flaviviruses, ten are from the partitioned datasets of flaviviruses, and 11 are from the partitioned datasets of the combined four genera of *Flaviviridae*, as shown in Table 1. We selected the ML tree from a complete and partitioned matrix aligned with a translation-based method as our hypothesis. We chose this alignment strategy as it maximized overall sequence identity and minimized the alignment length. We made all matrices and tree search results available in TreeBase (www.treebase.org, see Results section).

For the final hypothesis from ML, we executed the Shimodaira–Hasegawa approximate likelihood ratio test (SH-aLRT) as a measure of support [36] and also calculated clade frequencies using the ultrafast bootstrap method [37]. The SH-aLRT is an approximation of the likelihood ratio, a direct measure of how much the evidence supports the hypothesis. SH-aLRT is an approximation of the ratio of the log-likelihood of the optimal hypothesis and the best contradictory hypothesis. The ultrafast bootstrap analysis is a variation of the traditional bootstrap using heuristics and constraints to speed the search for optimal tree topologies, and the specialized literature shows that it is largely consistent with the traditional bootstrap analysis [37].

### 2.6. Sensitivity Analysis

We pooled all 31 cladograms, pruning off outgroup branches when necessary, to calculate the pairwise distance matrix among the phylogenetic hypothesis of flaviviruses based on match-split distances (MSdist) with the program MSdist v1.0 [38]. The match-split distances focus on the topological distance of unrooted phylogenetic trees based on splits, similarly to the Robinson-Foulds metric (RF; [39]). However, it is more sensitive and resistant than RF and to single terminal displacements. An original R script [40] was used to create a Sammon’s projection summarizing the distances among all tree topologies with the package “scamof” (available at https://cran.r-project.org/web/packages/smacof/index.html), which implements different approaches for multidimensional scaling [41]. A Sammon’s projection is a nonlinear projection method to map a high dimensional space onto a space of lower dimensionality. It is useful to simplify descriptions, summarize large data sets, recognize patterns, and visualize the class distribution [41]. An original R script was used to create a dendrogram and distance plot summarizing the distances among all tree topologies (Figure S1, Supplementary Materials).

Finally, we evaluated the sensitivity of optimality criteria to outgroup selection and data partitioning. Since we cannot directly compare the score of trees from different methodologies or datasets, we removed any terminals from the trees in Table 1 that were not shared among all trees. We also removed those terminals from the matrix used on the calculation of tree no. 0. We recalculated the likelihood scores using IQ-Tree.

### 2.7. Long-Branch Attraction Analysis

Felsenstein [42] introduced the issue of LBA in phylogenetics as a problem of statistical inconsistency that affects P. Felsenstein [42] advocated ML as an alternative. The issue becomes apparent “when two nonsister branches are long while other branches are short” (see [43], p. 202, for a historical perspective). However, there are also numerous accounts of matters of similar nature (e.g., long-branch repulsion) affecting ML, showing that it can also be inconsistent under certain conditions (e.g., [44,45,46]).

Authors such as Thézé et al. [17] (p. 2998) have raised concerns on possible effects of long branches from the outgroup, choosing to avoid the inclusion of terminals outside the ingroup altogether: “Midpoint rooting was chosen to root ML trees in order to avoid long-branch attraction with highly divergent outgroups.” Therefore, we evaluated the potential influences of long-branches on the hypothesis where we included the four genera of *Flaviviridae*.

We selected six terminals on our final topology that were visibly longer than all others and removed them, one at a time, from the complete dataset. This resulted in six additional matrices that we analyzed with TNT since P is more likely than ML to be affected by LBA. We used an original Python v3.6 script to prune all these terminals from every cladogram and applied MSdist to calculate the distance among all trees. Finally, we performed the Kruskall–Wallis test (a nonparametric approach to the one-way ANOVA) to evaluate the differences in the variance among different groups of trees.

### 2.8. Character Categorization

We carried the categorization of synapomorphies with YBYRÁ [47] to evaluate important mutations on clades of particular epidemiological interest. Synapomorphy refers to the shared occurence of a derived (apomorphic) character-state in a monophyletic group, no matter whether or not the character-state is homoplastic [48,49]. YBYRÁ indicates if a derived state occurs only in the clade in question (non-homoplastic) or also occurs in other clades (homoplastic) and if it is shared by all terminals of the clade (unique) or is subsequently transformed into one or more different states within the clade (non-unique). Homoplasy is error or incongruence in the codification of characters. Homoplasious character-states do not, by definition, identify the same historical entity. These characters have a non-minimal number of transformations on a tree [49]. Character categorization is useful to both support and describe clades. The YBYRÁ’s wiki page on GitLab contains additional a handful glossary for the most important phylogenetic concepts and terminology used here (visit https://gitlab.com/MachadoDJ/ybyra/-/wikis/Glossary).

### 2.9. Computational Resources

We performed all annotation and tree search in Heket, a high-memory server housed in the Museum of Zoology of the University of São Paulo (see http://www.ib.usp.br/grant/anfibios/researchHPC.html). Heket has a dual Processor Intel Xeon E52620v2 (24 cores), 256 GB DDR3 ECC, 4 × HDD 4 Tb (10,8 Tb RAID 5.0), SSD 240 GB, and Infiniband (20 Gb/s). It runs on an Ubuntu Linux v16.04 OS. We used a MacBook Pro (macOS High Sierra 10.13, 2.4 GHz Intel Core i5, 16 GB RAM) for alignment manipulation and analysis of results.

## 3. Results

### 3.1. Misalignments Are Frequently Observed When Data Is Not Partitioned

Clade 1 (*Aedes* spp. associated MBV I) shares 13 genes, of which seven were misaligned in the nucleotide MSA and seven were misaligned in the amino acid MSA. Clade 2 (*Aedes* spp. associated MBV II) shares 14 genes, of which four were misaligned in the nucleotide MSA and four were misaligned in the amino acid MSA. Clade 3 (ISFV-like I) shares 14 genes, of which two were misaligned in the nucleotide MSA and four were misaligned in the amino acid MSA. As expected, gene misalignment was even more frequent in the MSA of sequences from diverse flaviviruses. In this case, there were 14 shared genes, of which eight were misaligned in the nucleotide MSA and ten were misaligned in the amino acid MSA.

### 3.2. High Efficiency in Genome Annotation

After the removal of outliers by the Tukey’s method, the pipeline retained 118 complete genomes of *Flaviviridae* (Table S1, Supplementary Materials). We identified the soybean cyst nematode virus 5 (SbCNV-5) as a single outlier in our dataset (NCBI’s accession number NC_024077). The resulting genomic data comprises 1.25 Mbp (approx. 10,591 bp per genome).

With our pipeline, we re-annotated 63 reference genomes (from NCBI’s RefSeq) and 55 genomes (from NCBI’s RefSeq or GenBank) for which no annotations were available (File S1, Supplementary Materials). Although we developed the pipeline on a high-memory server, it is capable of processing the 119 genomes of the four genera of *Flaviviridae* on a personal computer in less than 2 h. Annotations sum up to 1247 mature peptides. During manual curation, we were required to perform editing in only 7% of the cases, which corresponded to short fragments such as 2k.

**Figure 3:**
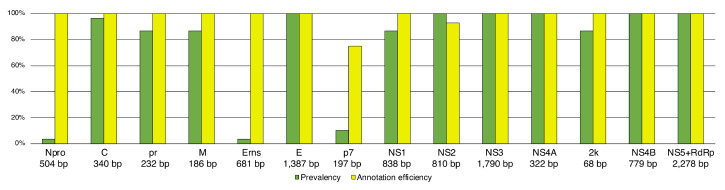
Efficiency of annotation pipeline and partition prevalence. The calculation of efficiency and prevalence considers 63 fully annotated reference sequences from the genera *Flavivirus* (48 sequences), *Hepacivirus* (seven sequences), *Pegivirus* (four sequences), and *Pestivirus* (four sequences). Prevalence is a measure of how widespread the partition is among the 63 annotated reference genomes. Annotation efficiency is a measure of the number of correct annotations when comparing the results with the 63 fully annotated reference genomes included in this study. There were no false positives. Values below each partition indicate their average length among the selected taxa.

The annotation efficiency for the 63 annotated genomes was of 100% for Npro, C, pr, M, Erns, E, NS1, NS2, NS3, NS4A, 2k, NS24B, and NS5+RdRp, independently of the genera. The annotation efficiency for annotated reference genomes was below 100% for NS2 (93%) and p7 (75%). Our pipeline did not recover the NS2 partition in *Pestivirus* genomes, but had 100% efficiency in the remanining genomes. We failed to recover the p7 gene in 4 of 5 sequences of *Pegivirus* but recover it in all genomes of *Pestivirus* and *Hepacivurus* (this partition does not occur in *Flavivirus*). It is possible that the relatively low efficiency to anotate the p7 gene is due to its short sequence length and small prevalence (Figure 3).

All gene partitions were recovered in the 55 non-annotated genomes we selected. The total efficiency of annotation considering the complete dataset of 118 genome sequences was 100% for *Flavivirus*, 100% for *Hepacivirus*, 89.2% for *Pegivirus*, and 88.89% for *Pestivirus* (see Table S4, Supplementary Materials).

The pipeline (which includes all original scripts) is available at https://gitlab.com/MachadoDJ/FLAVi under a GNU General Public License (GPL) v3.0.

### 3.3. Web Application

**Figure 4:**
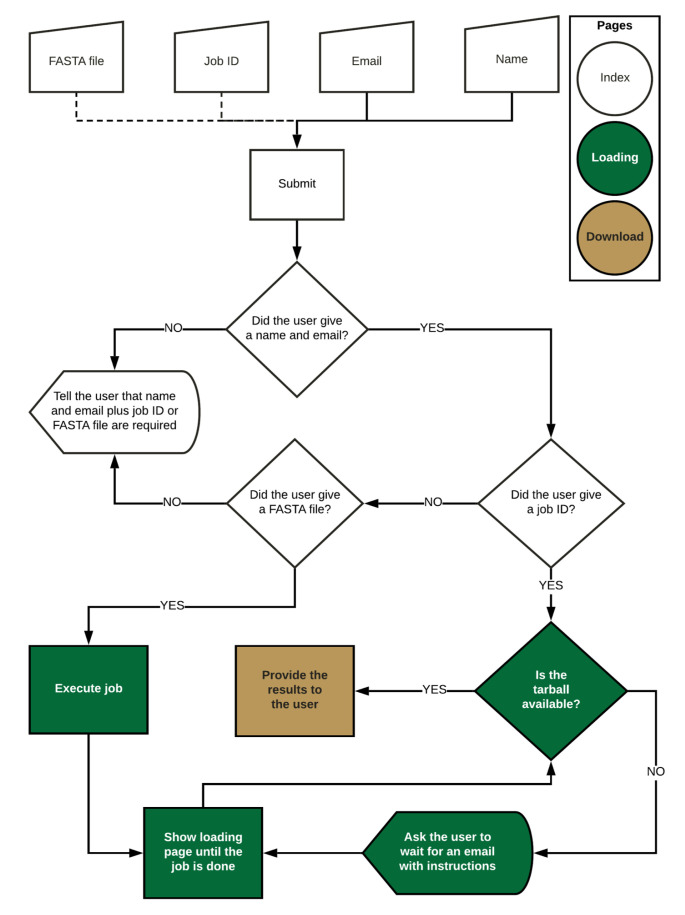
Main steps of the FLAVi-Web interface pipeline.

The web application is available at flavi-web.com. The main steps of the FLAVi-Web application are described in Figure 4. FLAVi-Web was created using Flask [50], a Python-based application for the creation of web interfaces. Flask uses HTML and JavaScript to create the aesthetic properties of the web interface. FLAVi-Web was first tested on macOS Mojave with 6 CPUs and 8GB RAM. The beta version was also tested on Ubuntu with 4 CPUs and 4GB RAM. A FASTA file with the virus sequences is annotated through the FLAVi pipeline in the background to produce a tar file with the main annotation results, including the gene annotation table in GFF3 format (see software.broadinstitute.org/software/igv/GFF). The additional files, found in the compressed folder are supplementary data about each step of the annotation pipeline. These can be used for further referencing applications. During testing, a multi-FASTA file of five sequences takes about 33 min to annotate and time increases based on File Size in a linear fashion.

The accuracy and outlier tests that are part of the FLAVi pipeline are replicated within FLAVi-Web. At the time of testing, there were few annotated genomes for *Flaviviridae*. In NCBI’s GenBank and RefSeq there were about 8860 complete genomes (until March 2020). We have found that only 5325 of these genomes were annotated (fully or partially). This results in  60% of the genomes of *Flaviviridae* being annotated at any level. We hope that FLAVi-Web will provide annotation data to published and future genomes, adding to their usefulness in many fields of research.

### 3.4. Topological Distances Formed Two Clusters

**Figure 5:**
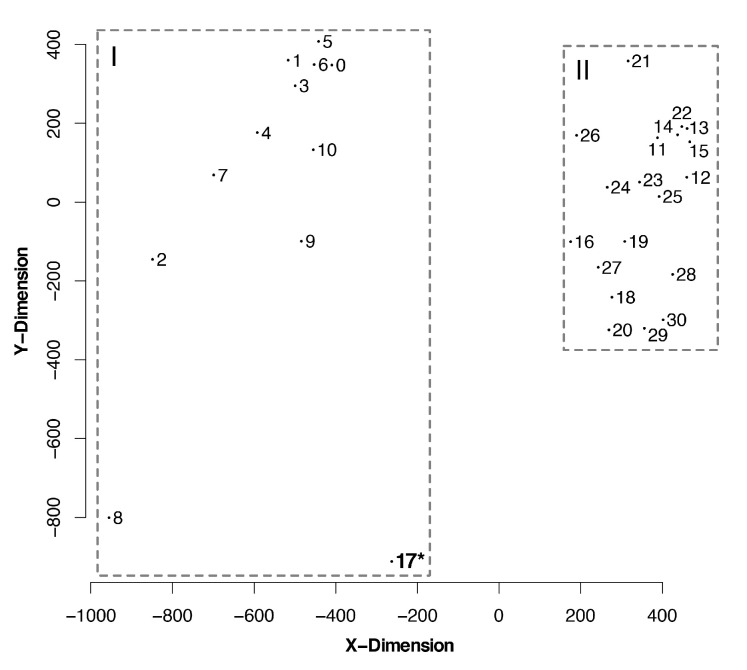
Sammon projection showing the multidimensional scaling of match-split distances among trees. The graphic displays the relationship among different tree topologies, clustering them into distinctive groups. Outgroup sequences (*Hepacivirus*, *Pegivirus*, and *Pestivirus*) were removed to guarantee the compared tree topologies would have the same terminals. Tree numbers correspond to those in Table 1. (**I**) Outgroup sequences and partitioned matrices. * This tree was produced without outgroup sequences. (**II**) No outgroup sequences; some matrices were partitioned. See Figure S1 (Supplementary Materials) for a dendrogram depicting the hierarchical clusters of trees based on match-split distances.

The matrix of match-split distances among unrooted binary topologies generated two clusters of trees (Figure 5). We did not observe clusters of trees based on optimality criteria or alignment strategy. That is to say that neither the optimality criteria nor the alignment methods seem to strongly direct tree search towards a specific portion of the tree-space.

However, we can categorize two distant clusters according to the presence (all *Flaviviridae*) or the absence (flaviviruses only) of outgroup sequences (groups I and II on Figure 5, respectively). The only tree topology obtained without outgroup sequences that was included on group II was a mostly parsimonious tree (No. 17; see Table 1) of flaviviruses, nested beside another most parsimonious tree from the same alignment and optimality criteria. These results show that there is a group of phylogenetic trees that are difficult to find without outgroup comparisons which, in phylogenetic analysis that includes different genera of *Flaviviridae*, are enabled by partitioned datasets. Furthermore, manual inspection of trees from the same optimality criterion revealed that clade support was improved when using partitioned datasets and outgroup comparisons (all matrices and trees are available at TreeBase, see Supplementary Materials).

### 3.5. The Scores Were Sensitive to Outgroup Selection and Data Partitioning

**Figure 6:**
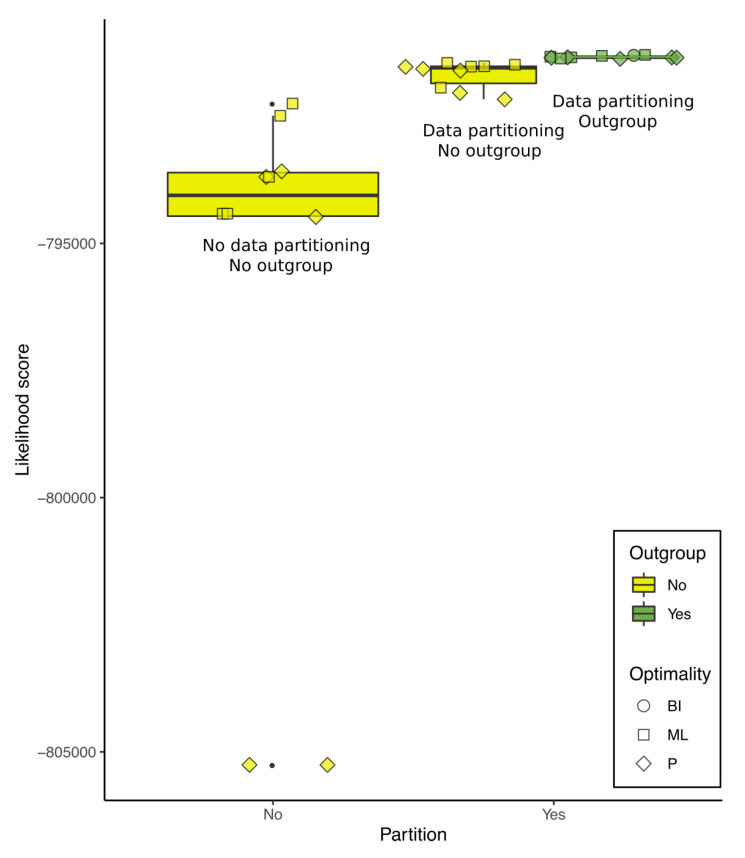
Likelihood scores of pruned trees calculated with IQ-Tree using the data from the matrix and partition scheme of the working hypothesis. The graphic shows that the likelihood scores are sensitive to data partitioning and outgroup selection. Tree numbers correspond to Table 1.

The optimality score was sensitive to outgroup selection and data partitioning in P, ML, and BI. To illustrate that, we pruned all trees from Table 1 so that they would have the same 102 terminals, and recalculated the likelihood scores of each of those trees with IQ-Tree using data from the same matrix and partition schemed as our working hypothesis (described below). The results are shown in Figure 6.

Each of the trees in Figure 6 represents the best heuristic solutions for their data and optimality criterion. Although this graphic serves to illustrate that scores are sensitive to outgroup selection and data partitioning, we caution the reader that we cannot completely detach the effects from outgroup selection from the gene annotation that makes it possible. Furthermore, although the working hypothesis has the highest score among the trees shown in Figure 6, we do not use this analysis to justify that it is better than any other. Instead, we based the working hypothesis on specific justifications for the choice of optimality criterion, alignment strategy, and the usage of as much available data as possible (see below).

### 3.6. No Long-Branch Distortions Were Observed

We compared the topological distances among 11 trees generated during LBA analysis, including three most parsimonious trees (MPTs) produced with total evidence (118 partitioned genomes, aligned using a translation-based method with PAM250 and analysed on TNT) and nine other trees generated when removing each of the six longest terminal branches in our dataset, one at a time: Tamana bat virus (NC_003996; one MPT), simian pegivirus (NC_024377; one MPT), equine pegivirus 1 (NC_020902; one MPT), human pegivirus 2 (NC_027998; three MPTs), rodent pegivirus (NC_021154; one MPT), and Norway rat pegivirus (NC_025679; two MPTs).

Although LBA can affect P, we did not observe any two long nonsister branches within clades composed of otherwise short branches in any of the 11 cladograms listed above. We also did not observe any event in which outgroup sequences were attracted to the largest ingroup branch (Tamana bat virus).

The calculation of normalized match-split distances shows that the distance among trees from total evidence (original) and trees with one of the long branches removed (new) vary in similar ways. That is to say that long branches do not seem to cause any more variation than the variation observed among the most optimal trees.

**Figure 7:**
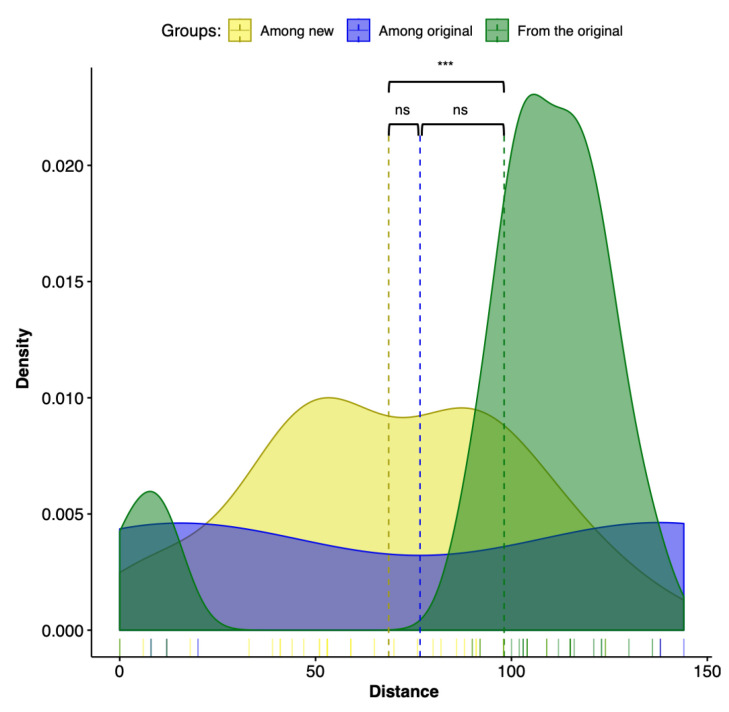
Density plot representing the distribution of the match-split distances among unrooted binary cladograms. The Kruskal–Wallis test was significant (*p* = 0.0048) only between groups “among new” and “from the original.” Dashed lines represent the mean of the groups.

The Kruskal–Wallis test was significant (*p* = 0.0048) only in one of the three comparisons: the variance of distances among new trees and the variance of all trees from the originals. These results are illustrated in the density plots, that use a kernel density estimate to show the probability density function of the variable. The density plots are a smoothed version of the histogram and is used in the same concept of Figure 7. The Kruskal–Wallis test results were expected, since our results indicate that the combination of sequence partitioning with an increased sample of outgroup sequences can impact the ingroup relationships.

### 3.7. An Updated Phylogeny of Flaviviridae

All 31 tree topologies and 15 matrices generated as a result of our evaluation of the potential impact of tree annotation and outgroup sampling over tree topology are available in TreeBASE (purl.org/phylo/treebase/phylows/study/TB2:S24096). As the hypothesis, we selected the tree topology from a complete and partitioned matrix aligned with the translation-based method and selected under the maximum likelihood (ML) criteria (tree No. 0 on Table 1). We chose ML as our preferred tree-building method given its relatively simple implementation and its ability to incorporate explicit models of molecular evolution while maintaining robustness in the face of differences in base composition and models of nucleotide substitution [51].

**Figure 8:**
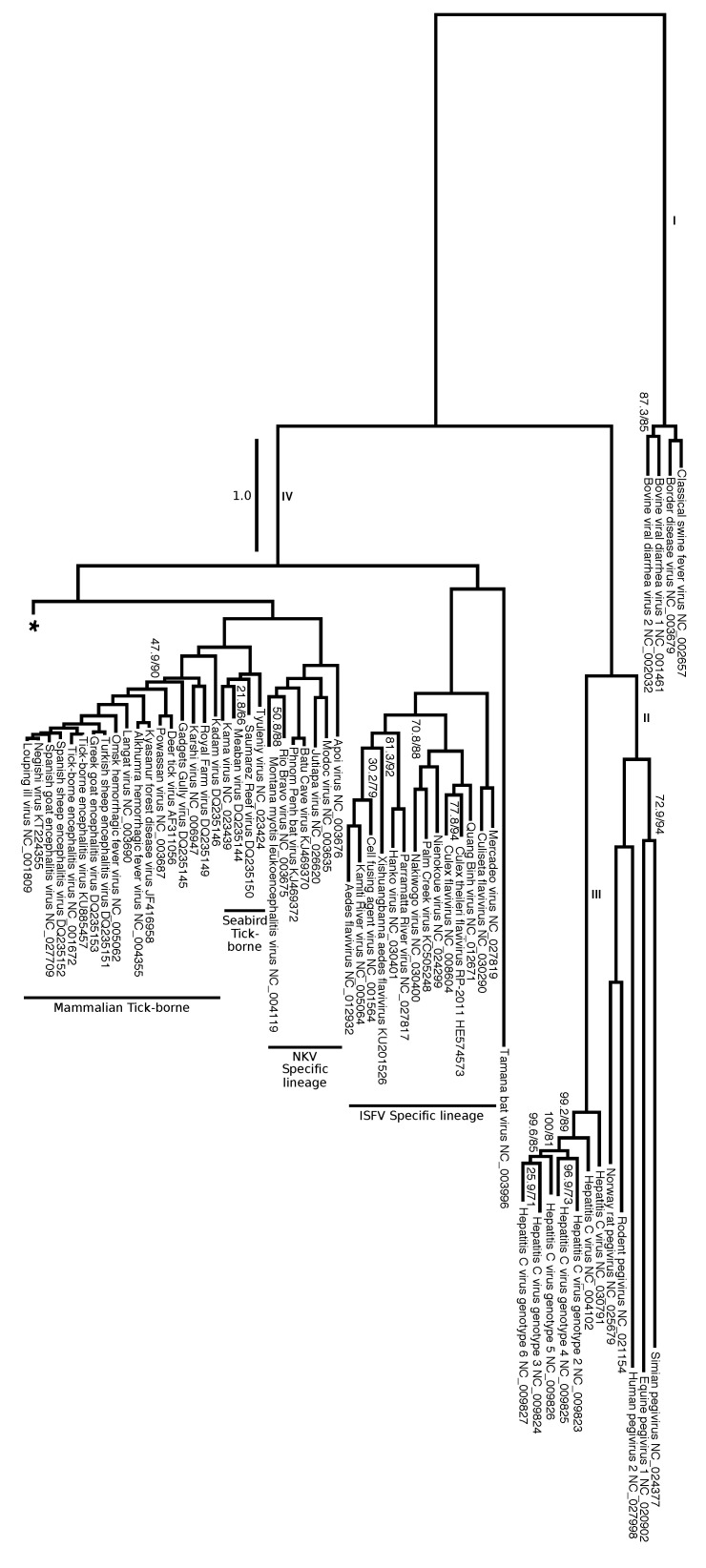
Phylogenetic hypothesis (tree No. 0 in Table 1). Branch lengths represent an estimation of the average number of nucleotide substitutions per site. Node labels indicate SH-aLRT support/ultrafast bootstrap (only shown if one of the values is below 90%). Clade names correlate to the character categorization analysis (Table S2, Supplementary Materials). Branch labels represent the four genera: I = *Pestivirus*; II = *Pegivirus*; III = *Hepacivirus*; IV = *Flavivirus*. Tree continues on Figure 8 (indicated by *). See Figure S2 (Supplementary Materials) for a full version of this tree.

**Figure 9:**
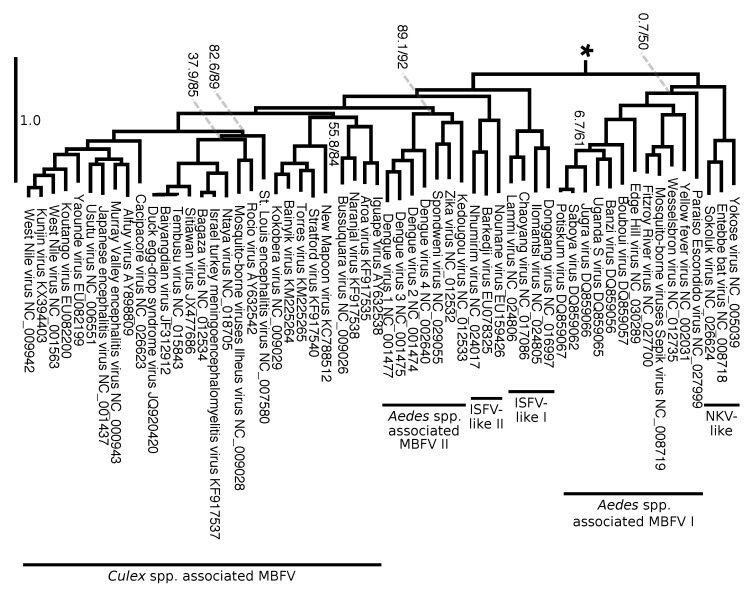
Continuation (from *) of the working phylogenetic hypothesis of *Flaviviridae* (see tree No. 0, Table 1, Figure 8). Branch lengths represent an estimation of average the number of nucleotide substitutions per site. Node labels indicate SH-aLRT support/ultrafast bootstrap (only shown if one of the values is below 90% too improve visualization). This image shows a group within flaviviruses which is sister to (NKV Specific lineage + (Seabird Tick-borne + Mammalian Tick-borne)). Δ = The Ecuador Paraiso Escondido virus (EPEV) was isolated from sand flies (*Psathyromyia abonnenci*). The EPEV was the first sand fly-borne flavivirus identified in the New World. See Figure S2 (Supplementary Materials) for a full version of this tree.

We split the evolutionary tree of *Flaviviridae* into two figures. Figure 8 focuses on the genera *Hepacivirus*, *Pegivirus*, and *Pestivirus* as well as on their relation to *Flavivirus*. Figure 9 presents the relationships among flaviviruses mostly related to tropical diseases transmitted by *Aedes* spp. and *Culex* spp., including DENV, WNV, YFV and ZIKV. It is interesting to note that there are a total of 11 branches that have an overall estimated number of substitutions per site greater than 1.0. However, the expected substitution rate per site varies across different positions on the alignment, and there are sufficient positions with low substitution rates that offer enough phylogenetic information for the estimating phylogenetic relationships within Flaviviridae. Long branches have branch support (measured by SH-aLRT) and frequencies (measure by ultrafast bootstrap) of 100, and most of the phylogeny contains clades that support and frequency values above 90% (with exceptions indicated on Figure 8 and Figure 9).

Overall, the tree shows high support and clade frequency values for most clades and corroborated the major groups of *Flavivirus* as presented by Moureau et al. [18]. The insect-specific flaviviruses (ISFV specific lineage), a group that infects only insects, was recovered as sister group to the remaining flaviviruses. All flaviviruses except the ISFV-specific lineage can be divided into nine clades: no known vector (NKV) specific lineage, seabird tick-borne, mammalian tick-borne (Figure 8), NKV-like, *Aedes* spp. associated mosquito-borne flaviviruses (MBFV) I, ISFV-like I, ISFV-like II, *Aedes* spp. associated MBFV II, and *Culex* spp. associated MBFV (Figure 9).

Similarly to what was observed by Moureau et al. [18], our analysis shows that three flaviviruses (Sokoluk, Entebbe bat, and Yokose virus) appear to have diverged within the mosquito-borne flaviviruses (MBFV) and lost their mosquito association completely, which justify entitling this clade NKV-like group. The NKV-like viruses are sister to the *Aedes* spp. associated MBFV I group, and both comprise a clade that is the sister group to the remaining flaviviruses, including ISFV-like and MBFV clades (Figure 9).

Our results also agreed with the phylogenetic analysis of Moureau et al. [18] on finding the ISFV-like lineages I and II to be paraphyletic. The ISFV-like II is the sister group to the clades *Aedes* spp. associated MBFV II and *Culex* spp. associated MBFV (Figure 9).

Despite agreeing to the principal clades in Moureau et al. [18] and the relationships among them, our analysis brings different insights to some phylogenetic relationships within each of them. We draw attention particularly to the internal relations of the clade *Aedes* spp. associated MBFV II in Figure 9, which places DENV and ZIKV in a much closer relationship than the one observed in Moureau et al. [18]. Although we cannot claim that our results represent the correct phylogeny of *Flaviviridae*, the size and diversity of our alignment matrix, together with the methodologies introduced here, makes it worthy of consideration together with other phylogenetic hypotheses. Moreover, we  show that genome annotation using FLAVi allows partitioned analysis with outgroup comparison, which results in the hypothesis that is mostly consistent with the specialized literature on *Flaviviridae*, but that could also bring different perspectives into the evolution of these viruses.

## 4. Discussion

### 4.1. Msas of Non-Partitioned Genome Sequences Can Be Misleading

Alignments of non-partitioned nucleotides or amino acids that comprise the polyprotein of flaviviruses hampers our ability to analyze the evolution of its individual genes. As observed, both amino acid and nucleotide MSAs will often lead to molecules from different genes aligning together. We demonstrated this by aligning a few sequences from phylogenetically related viruses. Nevertheless, nucleotide and amino acid misalignments will increase the more divergent the genomes are to each other.

It is not surprising that complete genome alignments will often lead to erroneous gene predictions. That is to say that aligning a non-annotated genome to a reference sequence and transferring gene predictions from the reference sequence to the non-annotated genome will not necessarily result in the correct gene predictions. Therefore, to produce MSAs that can can be used in the phylogenetic analysis of viruses, we must first annotate the genomes and then align the homologous sequences to each other. In this sense, FLAVi (https://gitlab.com/MachadoDJ/FLAVi) permits better alignments because it allows MSAs of individual nucleotide or amino acid sequences instead of the whole polyprotein. Additional discussion about the importance of data partitioning in phylogenetic systematics is beyond the scope of this manuscript but can be found in the specialized literature (e.g., [52,53,54,55]).

### 4.2. Flexible and Conservative Genome Annotation

A central goal of genetics is understanding how nucleotides encode complex biological functions, and genome annotation is a crucial step towards attaining this goal. In this manuscript, we took a conservative approach to annotating the genomic sequences so as to reduce false positives and minimize improper annotation of highly divergent sequences. The pipeline identified SbCNV-5 as the only outlier in our analyses. Upon careful examination, we observed that the SbCNV-5 genome length is 19.199 bp, approximately 46% larger than the largest genome size expected for *Flaviviridae*. When Bekal et al. [56] described SbCNV-5, they assigned it to *Flavivirus* after presenting protein homology to *Pestivirus*, citing that SbCNV-5 has genome structure, sfRNAs and viral maturation similar to flaviviruses. Nevertheless, the authors mention that SbCNV-5 has an enveloped spherical shaped virion that resembles flaviviruses, but larger (80 nm of size, compared to 50 nm in flaviviruses). The authors explain the difference in size to relate to the larger size of its genome in comparison to the “classical” genome of *Flaviviridae* (but see [25]). Although we cannot exclude the hypothesis of SbCNV-5 to share deep ancestry with the current genera of *Flaviviridae* viruses, we cannot place it within flaviviruses or any other of the current genera of *Flaviviridae*. In spite of the fact that we cannot infer the phylogenetic relationships of SbCNV-5 from our analysis due to its high dissimilarity to the members of *Flaviviridae* included here, this scenario could indicate that SbCNV-5 could land as a new genus of this family having as one of its characteristics a larger genome. It is also possible, however, that SbCNV-5 will become the link to a new family of viruses. More data on relatives of SbCNV-5 will be useful to test these scenarios.

### 4.3. Outgroup Comparison and Lba

Current phylogenetic strategies for genera of *Flaviviridae* often rely on the midpoint rooting of evolutionary trees. Authors are often driven towards midpoint rooting by the perceived difficulty of establishing a correct outgroup to act as root and the possibility of long-branch distortions, especially LBA [17,20,57].

The concern about branch distortions when including outgroup sequences in phylogenetic analysis is justifiable for all optimality criteria in cladistics (parsimony, maximum likelihood, or posterior probabilities calculated for Baeysian inferences). Although LBA was originally described by Felsenstein [42] as a bias in parsimony analysis, the specialized literature have demonstrated that likelihood and Bayesian inference are not immune to it [58]. Moreover, we now know that likelihood may also be affected by long-branch distortions, including long-branch repulsion (LBR), in which a true long-branch is not recovered [59]. However, the concern with LBA should not impede outgroup comparison when outgroups are available.

Outgroup comparison serves to root the topology and polarize character transformations [60,61]. This comparison is required to convert a network of abstract connections into a concrete evolutionary hypothesis [62]. Outgroup comparison also serves as a test of nested hypotheses of ingroup topology and homology [63,64].

Furthermore, empirical studies have demonstrated that increasing outgroup sampling may impact the phylogenetic relationship with ingroup terminals, adding support to clades that otherwise would not be recovered [23,65]. Thus, the relevance of outgroup comparison in phylogenetic systematics justifies the effort of dealing with possible long-branch distortions.

Since the specialized literature introduced the first empirical examples of LBA, we have access to different criteria to try to assess whether LBA could have affected the analysis or not [66]. These strategies are often based in observing well-supported clades of sufficiently long-branches within groups that otherwise include short branches. We found no instances of LBA in our dataset. Furthermore, the removal of one long-branch from the tree and the observation of its effect on tree topology could also be an indication of distortions caused by them. Still, pruning long-branches from the trees and calculating the topological distances among them does not show any indication of LBA.

Thus, we conclude that LBA did not affect the final phylogenetic hypothesis presented herein and that future research can rely on the same strategies employed here if authors are interested in locating possible LBA in their analysis. Nonetheless, it is worth noting that, since the real history of a group is not known, we cannot guarantee that long-branches do not represent legitimate sister taxa.

As noted by Wheeler [67], if we include a pair of *Panorpa* species (commonly known as scorpionflies) into an analysis of insect orders [68], the edge joining these taxa would show a great deal of change given the number of distinct transformations accumulated in the group. Nevertheless, there would be little reason to doubt that scorpionflies compose a monophyletic group.

### 4.4. Previous Work on the Phylogeny of Flavivirus

Before 2012, the phylogeny of *Flavivirus* did not include the ISFV-Specific Lineage, and mainly relied on utilizing the Cell Fusing Agent (CFA) as the outgroup of choice given its genetic distance to the other viruses of the genus [69,70,71,72]. Cook et al. [73] introduced a comprehensive phylogenetic study where it included multiple insect-specific viruses that form together a monophyletic group, which includes CFA, as a sister group to all other *Flavivirus*. These authors also performed an analysis where they included individual sequences from other genera of *Flaviviridae*, *Hepacivirus* and *Pestivirus* and find *Hepacivirus* as a sister group to *Flavivirus*, and *Pestivirus* sister to both *Flavivirus* and *Hepacivirus*. Our results are in agreement with these authors in regards to the main clades positioning on the trees, although the increase in the number of taxa has added more information within each vector based  group.

Moreover, Zanotto et al. [74] and Twiddy et al. [75] suggested that tick-borne and mosquito-borne flaviviruses have distinct population dynamics. These differences, mainly due to their methods of dispersal, propagation, and changes in the size of the host population, explain the difference on the branching process observed on tick-borne compared to mosquito-borne viruses (Figure 8 and Figure 9).

### 4.5. Phylogenetic Insights

Multiple researchers evaluated the phylogeny of *Flavivirus* using whole genomes. Although, when researchers wanted to increase taxon sampling, the lack of complete genomes available forced researchers to focus on E, NS3, and NS5 genes [69,71]. More recently, multiple groups attempted to generate a phylogeny of *Flavivirus* using the whole polyprotein information [18,76,77,78]. Our results are in agreement with most studies, except one. Li et al. [78] attempted an alignment-free method based on natural vectors and by doing so have found the tick-borne group sister to all other groups, including the ISFV-specific lineage. These authors present an alternative tree based on multiple sequence alignment of the whole polyprotein and tree building using neighbor-joining, where they could recover ISFV-specific lineage as the sister group to all other flaviviruses, but by doing so, they also find Tamana bat virus (TABV) as a distant sister-group to all flaviviruses.

Appropriate genome annotation allows the alignment of specific gene partitions, making it feasible to deal with variations in gene content and different levels of sequence divergence. Thus, we are capable of including more information into a phylogenetic framework that maximizes the explanatory power of the analysis and allows us to focus not only on the relationships within genera but also relationships between genera. When aligning whole polyproteins, multiple researchers left out the concept that each protein within a polyprotein is a modular unit. In doing so, the current phylogenetic studies assume homology of the whole polyprotein [76,78], although still assuming homology some take post-alignment processing steps for cleaning spurious regions before the phylogenetic analysis [18,77]. Ignoring the homology of individual proteins leads to the generation of spurious alignments and errors which will be perpetuated in downstream analysis [79].

Our novel annotation pipeline will allow better supported phylogenetic hypothesis for *Flaviviridae*. The addition of new sequences to the phylogeny based on homology statements may assist on the taxonomy and official classification of the viruses as part of *Flaviviridae* by groups such as the ICTV.

For instance, similarly to the SbCNV-5, the TABV (NC_003996) is also considered highly divergent from most flaviviruses. De Lamballerie et al. [80] mentions that they could not place TABV in a precise region on the tree, possibly due to the significant genetic differences from other members of the family. Thus, the authors suggested assigning TABV as a new genus. Moureau et al. [18] also considered TABV too divergent to be included given their particular methodology. Due to the partitioning of the data set according to the genes annotated with our pipeline, we were capable of including TABV into a phylogenetic framework that, despite its accumulation of genetic transformations, places it within *Flavivirus*. Specifically, TABV is placed as the sister group of the ISFV clade with high support values.

Within *Flavivirus*, we also observe that DENV forms a clade with ZIKV, Kedougou and Spondweni viruses, all of which are transmitted by *Aedes* spp. mosquitoes. Previously, Moureau et al. [18] identified these three viruses in a sister group to DENV, which encompass the *Culex* spp. associated group and previous related research suggested that the positioning of these viruses was ambiguous [81]. Nevertheless, individual studies focusing on specific viruses have observed this relationship [82]. Our results not only added to *Flaviviridae* phylogenetics but, while doing so, agreed with the current literature where we were able to recover the vast majority of known clades that share vector specificity. These results serve to bring additional details into the evolution of specific lineages of flaviviruses at the same time they demonstrate the ability of the methods presented here to deal with the diversity of genomes of *Flaviviridae*, including highly divergent sequences.

### 4.6. Final Remarks

The new approach presented here is a dedicated annotation pipeline for *Flaviviridae*. Our annotation pipeline uses a combination of ab initio and homology-based strategies and recovered 100% of the genes in *Flavivirus* and *Hepacivirus* genomes. In *Pegivirus* and *Pestivirus*, annotation efficiency was 100% except for one partition each. In *Pegivirus*, the annotation efficiency for p7 was only 20%. In *Pestivirus*, FLAVi failed to recover the NS2 gene (0%). There were no false positives. So far, the pipeline was extensively tested for *Flavivirus* and show promising results for the limited number of sequences of *Hepacivirus*, *Pegivirus*, and *Pestivirus* that we examined, with no false-positive results. The pipeline is available at gitlab.com/MachadoDJ/FLAVi. A web application is available at flavi-web.com.

The annotation of the genomes of *Flaviviridae* allows the combined phylogenetic analysis of all genera of the family (*Flavivirus*, *Hepacivirus*, *Pegivirus*, and *Pestivirus*). Within the universe of the phylogenetic analyses we performed here, the topology of *Flavivirus* was influenced by the inclusion of outgroup sequences from other genera of *Flaviviridae* for all cladistic optimality criteria (parsimony, maximum likelihood, and posterior probability). Additionaly, since the addition of outgroup sequences causes changes through the alignment, resulting in variations of character statements (i.e., the proposition of homologous characters and their character states), we cannot completely detach the effects of outgroup selection from the effects of the data partitioning that make it possible. The inclusion of outgroup sequences did not result in any noticeable long-branch distortions.

The final tree sheds light on the phylogenetic relationship of viruses that are divergent from most *Flaviviridae*. The newest release of the virus taxonomy and the International Code of Virus Classification and Nomenclature ratified by the ICTV does not list SbCNV-5 as a *Flavivirus* and lists TABV as unclassified, but related to flaviviruses [83]. Our results indicate that the SbCNV-5 may not be a member of *Flaviviridae*, and that TABV belongs to *Flavivirus* and is especially closely related to insect-specific flaviviruses (ISFV specific lineage). We also corroborate the close phylogenetic relationship between DENV and ZIKV. The main phylogenetic insights of our phylogeny reconstruction were made possible due to the inclusion of all genera of *Flaviviridae* into a single analysis which maximized the explanatory power of the data and improved the resolution of phylogenetic relationships in the ingroup considering all the tree search experiments we performed.

## Figures and Tables

**Table 1:** Enumeration of different tree search strategies. “Translation” and “Geneious MSA” indicates the translation-based alignment tool and the multiple sequence alignment tool provided in Geneious v8.1.9, respectively. The ditto mark (") indicates values that are the same as the above.

Tree No.	Partition	Group	Optimality Criteria	Tree Search Program	Alignment Strategy
0	Yes	*Flaviviridae*	Maximum likelihood	IQ-Tree	Translation (PAM250)
1	"	"	Bayesian inference	BEAST	Translation (PAM250)
2	"	"	Parsimony	TNT	Translation (PAM250)
3	"	"	Maximum likelihood	IQ-tree	Clustal
4	"	"	"	"	Geneious MSA
5	"	"	"	"	Mafft
6	"	"	"	"	MUSCLE
7	"	"	Parsimony	TNT	Clustal
8	"	"	"	"	Geneious MSA
9	"	"	"	"	Mafft
10	"	"	"	"	MUSCLE
11	"	*Flavivirus*	Maximum likelihood	IQ-Tree	Clustal
12	"	"	"	"	Geneious MSA
13	"	"	"	"	Mafft
14	"	"	"	"	MUSCLE
15	"	"	"	"	Translation (PAM250)
16	"	"	Parsimony	TNT	Clustal
17	"	"	"	"	Geneious MSA
18	"	"	"	"	Mafft
19	"	"	"	"	MUSCLE
20	"	"	"	"	Translation (PAM250)
21	No	"	Maximum likelihood	IQ-Tree	Clustal
22	"	"	"	"	Mafft
23	"	"	"	"	Translation (PAM100)
24	"	"	"	"	Translation (PAM200)
25	"	"	"	"	Translation (PAM250)
26	"	"	Parsimony	TNT	Clustal
27	"	"	"	"	Mafft
28	"	"	"	"	Translation (PAM100)
29	"	"	"	"	Translation (PAM200)
30	"	"	"	"	Translation (PAM250)

## Supplementary Materials

The supplementary material listed below are available at Zenodo (doi:10.5281/zenodo.3975978). Table S1 (tableS1.csv): genomic data. Table S2 (tableS2.csv): character categorization for selected nodes. Table S3 (tableS3.csv): programs and parameters. Table S4 (tableS4.csv): annotation efficiency per partition. File S1 (fileS1.gff): annotation. File S2 (fileS2.xml): configuration file for BEAST 2 (configuration.xml). Figure S1 (figureS1.pdf): dendrogram depicting the hierarchical clusters of trees based on match-split distances. Figure S2 (figureS2.pdf): full version of the working phylogenetic hypothesis (tree No. 0 in Table 1). In adition to the material listed above, all 31 tree topologies and 15 alignment matrices discussed in this manuscript are available in TreeBASE (purl.org/phylo/treebase/phylows/study/TB2:S24096). The FLAVi pipeline and all the original scripts are available at GitLab (gitlab.com/MachadoDJ/FLAVi). The web application can be accessed at flavi-web.com.

## References

[1] Simmonds P., Becher P., Bukh J., Gould E.A., Meyers G., Monath T., Muerhoff S., Pletnev A., Rico-Hesse R., Smith D.B. (2017). ICTV virus taxonomy profile: Flaviviridae. J. Gen. Virol..

[2] Thomas D.L., Astemborski J., Rai R.M., Anania F.A., Schaeffer M., Galai N., Nolt K., Nelson K.E., Strathdee S.A., Johnson L. (2000). The natural history of hepatitis C virus infection: Host, viral, and environmental factors. JAMA.

[3] Balcom E.F., Doan M.A., Branton W.G., Jovel J., Blevins G., Edguer B., Hobman T.C., Yacyshyn E., Emery D., Box A. (2018). Human pegivirus-1 associated leukoencephalitis: Clinical and molecular features. Ann. Neurol..

[4] Houe H. (1999). Epidemiological features and economical importance of bovine virus diarrhoea virus (BVDV) infections. Vet. Microbiol..

[5] Holbrook M. (2017). Historical perspectives on *Flavivirus* research. Viruses.

[6] Hulo C., De Castro E., Masson P., Bougueleret L., Bairoch A., Xenarios I., Le Mercier P. (2010). ViralZone: A knowledge resource to understand virus diversity. Nucleic Acids Res..

[7] Clark K., Karsch-Mizrachi I., Lipman D.J., Ostell J., Sayers E.W. (2015). GenBank. Nucleic Acids Res..

[8] Charles J., Tangudu C.S., Hurt S.L., Tumescheit C., Firth A.E., Garcia-Rejon J.E., Machain-Williams C., Blitvich B.J. (2018). Detection of novel and recognized RNA viruses in mosquitoes from the Yucatan Peninsula of Mexico using metagenomics and characterization of their in vitro host ranges. J. Gen. Virol..

[9] Altschul S.F., Gish W., Miller W., Myers E.W., Lipman D.J. (1990). Basic local alignment search tool. J. Mol. Biol..

[10] Wu Z., Liu B., Du J., Zhang J., Lu L., Zhu G., Han Y., Su H., Yang L., Zhang S. (2018). Discovery of diverse rodent and bat pestiviruses with distinct genomic and phylogenetic characteristics in several Chinese provinces. Front. Microbiol..

[11] El-Gebali S., Mistry J., Bateman A., Eddy S.R., Luciani A., Potter S.C., Qureshi M., Richardson L.J., Salazar G.A., Smart A. (2018). The Pfam protein families database in 2019. Nucleic Acids Res..

[12] Mitchell A.L., Attwood T.K., Babbitt P.C., Blum M., Bork P., Bridge A., Brown S.D., Chang H.Y., El-Gebali S., Fraser M.I. (2018). InterPro in 2019: Improving coverage, classification and access to protein sequence annotations. Nucleic Acids Res..

[13] Marchler-Bauer A., Bo Y., Han L., He J., Lanczycki C.J., Lu S., Chitsaz F., Derbyshire M.K., Geer R.C., Gonzales N.R. (2016). CDD/SPARCLE: Functional classification of proteins via subfamily domain architectures. Nucleic Acids Res..

[14] Wen S., Ma D., Lin Y., Li L., Hong S., Li X., Wang X., Xi J., Qiu L., Pan Y. (2018). Complete Genome Characterization of the 2017 Dengue Outbreak in Xishuangbanna, a Border City of China, Burma and Laos. Front. Cell. Infect. Microbiol..

[15] Yachdav G., Kloppmann E., Kajan L., Hecht M., Goldberg T., Hamp T., Hönigschmid P., Schafferhans A., Roos M., Bernhofer M. (2014). PredictProtein: An open resource for online prediction of protein structural and functional features. Nucleic Acids Res..

[16] Liu L., Xia H., Wahlberg N., Belák S., Baule C. (2009). Phylogeny, classification and evolutionary insights into pestiviruses. Virology.

[17] Thézé J., Lowes S., Parker J., Pybus O.G. (2015). Evolutionary and phylogenetic analysis of the hepaciviruses and pegiviruses. Genome Biol. Evol..

[18] Moureau G., Cook S., Lemey P., Nougairede A., Forrester N.L., Khasnatinov M., Charrel R.N., Firth A.E., Gould E.A., De Lamballerie X. (2015). New insights into *Flavivirus* evolution, taxonomy and biogeographic history, extended by analysis of canonical and alternative coding sequences. PLoS ONE.

[19] Maddison W.P., Donoghue M.J., Maddison D.R. (1984). Outgroup analysis and parsimony. Syst. Biol..

[20] Hess P.N., De Moraes Russo C.A. (2007). An empirical test of the midpoint rooting method. Biol. J. Linn. Soc..

[21] Kinene T., Wainaina J., Maina S., Boykin L. (2016). Rooting trees, methods for. Encycl. Evol. Biol..

[22] Wenzel J. (2020). Origins of SARS-CoV-1 and SARS-CoV-2 are often poorly explored in leading publications. Cladistics.

[23] Grant T. (2019). Outgroup sampling in phylogenetics: Severity of test and successive outgroup expansion. J. Zool. Syst. Evol. Res..

[24] de Bernardi Schneider A., Malone R.W., Guo J.T., Homan J., Linchangco G., Witter Z.L., Vinesett D., Damodaran L., Janies D.A. (2017). Molecular evolution of Zika virus as it crossed the Pacific to the Americas. Cladistics.

[25] Shi M., Lin X.D., Vasilakis N., Tian J.H., Li C.X., Chen L.J., Eastwood G., Diao X.N., Chen M.H., Chen X. (2016). Divergent viruses discovered in arthropods and vertebrates revise the evolutionary history of the *Flaviviridae* and related viruses. J. Virol..

[26] Birney E., Clamp M., Durbin R. (2004). GeneWise and Genomewise. Genome Res..

[27] Haas B.J., Papanicolaou A., Yassour M., Grabherr M., Blood P.D., Bowden J., Couger M.B., Eccles D., Li B., Lieber M. (2013). *De novo* transcript sequence reconstruction from RNA-seq using the Trinity platform for reference generation and analysis. Nat. Protoc..

[28] Consortium T.U. (2018). UniProt: A worldwide hub of protein knowledge. Nucleic Acids Res..

[29] Rice P., Longden I., Bleasby A. (2000). EMBOSS: The European molecular biology open software suite. Trends Genet..

[30] Tukey J.W. (1977). Exploratory Data Analysis.

[31] Sievers F., Wilm A., Dineen D., Gibson T.J., Karplus K., Li W., Lopez R., McWilliam H., Remmert M., Söding J. (2011). Fast, scalable generation of high-quality protein multiple sequence alignments using Clustal Omega. Mol. Syst. Biol..

[32] Kearse M., Moir R., Wilson A., Stones-Havas S., Cheung M., Sturrock S., Buxton S., Cooper A., Markowitz S., Duran C. (2012). Geneious Basic: An integrated and extendable desktop software platform for the organization and analysis of sequence data. Bioinformatics.

[33] Goloboff P.A., Farris J.S., Nixon K.C. (2008). TNT, a free program for phylogenetic analysis. Cladistics.

[34] Nguyen L.T., Schmidt H.A., von Haeseler A., Minh B.Q. (2014). IQ-TREE: A fast and effective stochastic algorithm for estimating maximum-likelihood phylogenies. Mol. Biol. Evol..

[35] Bouckaert R., Heled J., Kühnert D., Vaughan T., Wu C.H., Xie D., Suchard M.A., Rambaut A., Drummond A.J. (2014). BEAST 2: A software platform for Bayesian evolutionary analysis. PLoS Comput. Biol..

[36] Guindon S., Dufayard J.F., Lefort V., Anisimova M., Hordijk W., Gascuel O. (2010). New algorithms and methods to estimate maximum-likelihood phylogenies: Assessing the performance of PhyML 3.0. Syst. Biol..

[37] Minh B.Q., Nguyen M.A.T., von Haeseler A. (2013). Ultrafast approximation for phylogenetic bootstrap. Mol. Biol. Evol..

[38] Bogdanowicz D., Giaro K. (2012). Matching split distance for unrooted binary phylogenetic trees. IEEE/ACM Trans. Comput. Biol. Bioinform..

[39] Robinson D.F., Foulds L.R. (1981). Comparison of phylogenetic trees. Math. Biosci..

[40] R Core Team (2014). R: A Language and Environment for Statistical Computing.

[41] Sammon J.W. (1969). A nonlinear mapping for data structure analysis. IEEE Trans. Comput..

[42] Felsenstein J. (1978). Cases in which parsimony or compatibility methods will be positively misleading. Syst. Zool..

[43] Farris J.S. (1999). Likelihood and inconsistency. Cladistics.

[44] Siddall M.E., Whiting M.F. (1999). Long-branch abstractions. Cladistics.

[45] Kück P., Mayer C., Wägele J.W., Misof B. (2012). Long branch effects distort maximum likelihood phylogenies in simulations despite selection of the correct model. PLoS ONE.

[46] Chang J.T. (1996). Inconsistency of evolutionary tree topology reconstruction methods when substitution rates vary across characters. Math. Biosci..

[47] Machado D.J. (2015). YBYRÁ facilitates comparison of large phylogenetic trees. BMC Bioinform..

[48] Grant T., Kluge A.G. (2004). Transformation series as an ideographic character concept. Cladistics.

[49] Nixon K.C., Carpenter J.M. (2012). On homology. Cladistics.

[50] Grinberg M. (2014). Flask Web Development: Developing Web Applications with Python.

[51] Huelsenbeck J.P. (1995). The robustness of two phylogenetic methods: Four-taxon simulations reveal a slight superiority of maximum likelihood over neighbor joining. Mol. Biol. Evol..

[52] Buckley T.R., Simon C., Chambers G.K. (2001). Exploring among-site rate variation models in a maximum likelihood framework using empirical data: Effects of model assumptions on estimates of topology, branch lengths, and bootstrap support. Syst. Biol..

[53] Nylander J.A., Ronquist F., Huelsenbeck J.P., Nieves-Aldrey J. (2004). Bayesian phylogenetic analysis of combined data. Syst. Biol..

[54] Brown J.M., Lemmon A.R. (2007). The importance of data partitioning and the utility of Bayes factors in Bayesian phylogenetics. Syst. Biol..

[55] Kainer D., Lanfear R. (2015). The effects of partitioning on phylogenetic inference. Mol. Biol. Evol..

[56] Bekal S., Domier L.L., Gonfa B., McCoppin N.K., Lambert K.N., Bhalerao K. (2014). A novel *Flavivirus* in the soybean cyst nematode. J. Gen. Virol..

[57] Wheeler W.C. (1990). Nucleic acid sequence phylogeny and random outgroups. Cladistics.

[58] Bergsten J. (2005). A review of long-branch attraction. Cladistics.

[59] Pol D., Siddall M.E. (2001). Biases in maximum likelihood and parsimony: A simulation approach to a 10-taxon case. Cladistics.

[60] Farris J.S. (1972). Estimating phylogenetic trees from distance matrices. Am. Nat..

[61] Farris J.S. (1982). Outgroups and parsimony. Syst. Zool..

[62] Lundberg J.G. (1972). Wagner networks and ancestors. Syst. Biol..

[63] Kluge A.G., Grant T. (2006). From conviction to anti-superfluity: Old and new justifications of parsimony in phylogenetic inference. Cladistics.

[64] Grant T., Kluge A.G. (2009). Perspective: Parsimony, explanatory power, and dynamic homology testing. Syst. Biodivers..

[65] Grant T., Rada M., Anganoy-Criollo M., Batista A., Dias P.H., Jeckel A.M., Machado D.J., Rueda-Almonacid J.V. (2017). Phylogenetic systematics of dart-poison frogs and their relatives revisited (Anura: Dendrobatoidea). South Am. J. Herpetol..

[66] Huelsenbeck J.P. (1997). Is the Felsenstein zone a fly trap?. Syst. Biol..

[67] Wheeler W.C. (2012). Systematics: A Course of Lectures.

[68] Wheeler W. (2001). Homology and the optimization of DNA sequence data. Cladistics.

[69] Kuno G., Chang G.J.J., Tsuchiya K.R., Karabatsos N., Cropp C.B. (1998). Phylogeny of the genus Flavivirus. J. Virol..

[70] Jenkins G.M., Pagel M., Gould E.A., Paolo M.d.A., Holmes E.C. (2001). Evolution of base composition and codon usage bias in the genus Flavivirus. J. Mol. Evol..

[71] Billoir F., de Chesse R., Tolou H., de Micco P., Gould E.A., de Lamballerie X. (2000). Phylogeny of the genus Flavivirus using complete coding sequences of arthropod-borne viruses and viruses with no known vector. J. Gen. Virol..

[72] Schubert A.M., Putonti C. (2010). Evolution of the sequence composition of flaviviruses. Infect. Genet. Evol..

[73] Cook S., Moureau G., Kitchen A., Gould E.A., de Lamballerie X., Holmes E.C., Harbach R.E. (2012). Molecular evolution of the insect-specific flaviviruses. J. Gen. Virol..

[74] Zanotto P.d., Gould E.A., Gao G.F., Harvey P.H., Holmes E.C. (1996). Population dynamics of flaviviruses revealed by molecular phylogenies. Proc. Natl. Acad. Sci. USA.

[75] Twiddy S.S., Pybus O.G., Holmes E.C. (2003). Comparative population dynamics of mosquito-borne flaviviruses. Infect. Genet. Evol..

[76] Lobo F.P., Mota B.E., Pena S.D., Azevedo V., Macedo A.M., Tauch A., Machado C.R., Franco G.R. (2009). Virus-host coevolution: Common patterns of nucleotide motif usage in Flaviviridae and their hosts. PLoS ONE.

[77] Huhtamo E., Cook S., Moureau G., Uzcátegui N.Y., Sironen T., Kuivanen S., Putkuri N., Kurkela S., Harbach R.E., Firth A.E. (2014). Novel flaviviruses from mosquitoes: Mosquito-specific evolutionary lineages within the phylogenetic group of mosquito-borne flaviviruses. Virology.

[78] Li Y., He L., He R.L., Yau S.S.T. (2017). Zika and flaviviruses phylogeny based on the alignment-free natural vector method. DNA Cell Biol..

[79] Springer M.S., Gatesy J. (2018). On the importance of homology in the age of phylogenomics. Syst. Biodivers..

[80] De Lamballerie X., Crochu S., Billoir F., Neyts J., De Micco P., Holmes E., Gould E. (2002). Genome sequence analysis of Tamana bat virus and its relationship with the genus Flavivirus. J. Gen. Virol..

[81] Gupta S.K., Singh S., Nischal A., Pant K.K., Seth P.K. (2014). Molecular-based identification and phylogeny of genomic and proteomic sequences of mosquito-borne *Flavivirus*. Genes Genom..

[82] Alkan C., Zapata S., Bichaud L., Moureau G., Lemey P., Firth A.E., Gritsun T.S., Gould E.A., de Lamballerie X., Depaquit J. (2015). Ecuador Paraiso Escondido virus, a new *Flavivirus* isolated from New World sand flies in Ecuador, is the first representative of a novel clade in the genus Flavivirus. J. Virol..

[83] Walker P.J., Siddell S.G., Lefkowitz E.J., Mushegian A.R., Dempsey D.M., Dutilh B.E., Harrach B., Harrison R.L., Hendrickson R.C., Junglen S. (2019). Changes to virus taxonomy and the International Code of Virus Classification and Nomenclature ratified by the International Committee on Taxonomy of Viruses (2019). Arch. Virol..

